# Potential immunogenic modulation of hypo-fractionated radiotherapy at optimal schedules and the subsequent vaccine-like effect of local irradiation - a systematic review

**DOI:** 10.3389/fonc.2025.1546875

**Published:** 2025-10-03

**Authors:** Jagtar Singh, Martin Ashdown, Siddhartha Baxi

**Affiliations:** ^1^ Mount Gambier & Districts Health Service, Limestone Coast Local Health Network Inc, Mount Gambier, SA, Australia; ^2^ School of Pharmacy and Medical Sciences, Griffith University, Southport, QLD, Australia; ^3^ Faculty of Medicine, The University of Melbourne, Parkville, VIC, Australia; ^4^ Genesis Care, John Flynn Hospital, Tugun, QLD, Australia; ^5^ School of Medicine, Griffith University, Southport, QLD, Australia

**Keywords:** hypo-fractionated radiotherapy, CD8+ T-cells infiltration, immune stimulation, abscopal effect, clinical outcomes

## Abstract

**Introduction:**

Hypo-fractionated radiotherapy (HFRT) regimens can induce immune system activation and help to identify a therapeutic window after RT by measuring cytotoxic T-cell concentration. Here, we summarise previous preclinical and clinical studies on the effects of HFRT on the immune system, both locally and systemically. We also investigate the existing data on the optimal dose and fractionation scheme of HFRT to enhance local and distant anti-tumour immunity.

**Methods:**

A search was conducted using the PubMed, ScienceDirect, and Google Scholar databases. The systematic review was conducted in accordance with the PRISMA-DTA guidelines. Quality was assessed utilising the Prediction model Risk Of Bias ASsessment Tool (PROBAST). Data from publications that met quality criteria were grouped via (1) hypo-fractionated radiotherapy, (2) CD8+ T-cells infiltration, (3), immune stimulation, and (4) abscopal effect.

**Results:**

After eligibility consideration, 12 studies (7 = preclinical and 5 = clinical) were selected for this systematic review article. Ten of the 12 studies observed T-cell infiltration into the tumour environment following HFRT. Moreover, six of 12 preclinical and clinical studies tested the HFRT schemes with several-day intervals to control tumour growth. To assess the possible immunogenic impact of HFRT on the immune system both locally and systemically, eight previous studies examined the abscopal effect (AE) and response rates following optimal HFRT schedules.

**Conclusions:**

Existing literature suggests that HFRT with an optimal regimen can induce the activation of T lymphocytes and break tumour tolerance while simultaneously reducing the frequency of Tregs. The collected studies also suggested that optimal dosages and fractions of HFRT induce an immune response. However, it should be further explored to provide clinicians with information that would be valuable when making decisions regarding patient care. This strategy may simplify protocols, increase cancer patients’ response rate to treatment, lower costs, and lower their chance of toxicity and developing immune-related side effects after receiving chemotherapy and immunotherapy.

## 1 Introduction

Radiation therapy (RT) is usually considered a “local” treatment modality in cancer therapy because radiation can only directly eradicate cancer cells within the radiation field. Because of recent developments in image guidance and RT delivery methods, single ablative high doses can be safely delivered to many tumour sites by using stereotactic radiosurgery (SRS), stereotactic body RT (SBRT), or stereotactic ablative body irradiation (SABR) (1–4). High doses of radiation can be achieved by a single treatment (extreme oligo-fractionation) or by 2 to 5 high-dose treatments (oligo-fractionation or hypo-fractionation), serving as an alternative to conventional daily low-dose fractionated treatments (<3 Gy) over several weeks (5). Limited results showed improved efficacy compared with traditional fractionated RT in managing advanced or metastatic colorectal, liver, and non-small cell lung tumours (2). The outcome can be comparable to surgery for resectable tumours, and SRS can be applied to unresectable tumours (2, 6).

Hypo-fractionated RT (HFRT) is a modern radiation technique that provides targeted high-dose irradiation to a tumour while limiting damage to surrounding normal tissues (7–9). HFRT directly kills tumour cells via DNA double-strand breaks and propagates dose-dependent vascular damage and destruction of the tumour microenvironment, causing secondary tumour cell death (10–12). Massive tumour cell death because of DNA damage and vascular injury functions can produce strong anti-tumour immunity. Therefore, it has been reported that the anti-tumour immune response plays a significant role in the outcome of SABR (10–12). However, RT may result in poor outcomes in patients with a weakened immune system, whereas it may effectively eradicate tumours in patients with a more robust immune system (13, 14).

It has been shown that RT may contribute to making tumours visible to the immune system (15–19). After RT treatment, MHC-I molecules display an increased pool of peptides for antigen presentation (20). Dendritic cells (DCs) can capture tumour-associated antigens (TAAs) released to the tumour periphery (21). These DCs become active via toll-like receptor (TLRs) recognition, in which endogenous danger signals emitted by dying tumour cells are identified (21). The activation of DCs is characterised by the upregulation of cell surface molecules involved in antigen presentation and co-stimulation (e.g., CD80 and CD86) and the release of pro-inflammatory cytokines (21). Thus, activated DCs migrate to secondary lymphoid organs, where TAAs are presented to CD4+ Th cells in the MHC-II context (21). Active, effector T-cells may recirculate through the body and generate a tumour-specific immune response in distant areas (21). Using this mechanism, adaptive immune responses may help to eradicate metastasis of tumours that do not express MHC-II. CD4+ T-cells may help kill tumour cells through several mechanisms (21). One such mechanism enables the development of tumour-specific CD8+ T-cells that recognise tumour peptides by MHC-I (21).

A growing body of evidence suggests that the systemic anti-tumour effect in metastatic disease in response to high-dose local radiation results in the regression of non-irradiated distant tumour sites (22). This phenomenon, known as the abscopal effect (AE) of radiation, was first described by RH Mole in 1953 (23). Multiple mechanisms have been proposed to cause the AE (16, 24), such as the systemic secretion of specific cytokines and chemokines, a systemic immune response against local tumour antigens released, or local inflammation that can lead to a distant effect (25). In any case, the hypothesis that the AE is immune-mediated is becoming stronger. If the radiation dose is sufficient to generate cell death, this can lead to the induction of the adaptive immune response. RT directly elicits an innate immune recognition of tumour by releasing danger signals”. Thus, these signals can increase immune-mediated cell death, which promotes the uptake of circulating tumour antigens by DCs via cross-priming and ultimately leads to the activation of tumour-specific T-cell response (26). The tumour-specific T-cells are capable of recirculating throughout the body, detecting any tumour cells (across multiple antigens) and eradicating them (24, 27). Therefore, tumours that are even at a distance from the irradiated field can be immunologically killed (24, 27). This is described as an AE (24, 27, 28).

Here, we summarise previous preclinical and clinical studies on the effects of hypo-fractionated RT (HFRT) on the immune system locally and systemically. We also investigate the existing data on the optimal dose and fractionation scheme of HFRT to enhance local and distant anti-tumour immunity.

## 2 Materials and methods

### 2.1 Search strategy and study selection

This systematic review followed the PRISMA statement for reporting systematic reviews and meta-analyses (29). A comprehensive electronic search was conducted between March and October 2024 using PubMed (https://pubmed.ncbi.nlm.nih.gov/), ScienceDirect (https://www.sciencedirect.com/), and Google Scholar (https://scholar.google.com.au/) databases for articles published between 2010 and 2024. The studies investigated RT-induced immune stimulation at optimal HFRT improves AE and clinical outcomes. The systematic search for relevant studies was carried out using the following keywords: RT, hypo-fractionation RT, immune system, anti-tumour CD8+ T, Infiltration of CD8+ cytotoxic T-cells, tumour-specific, monocytic myeloid-derived suppressor cells (M-MDSCs), immune stimulation, RT-schedule, RT-dose, RT-fraction, and clinical outcome. Similarly, we performed a manual review of references to select additional studies. **Table 1** summarises the search strategy of this systematic review.

**Table 1:** Summarises the search strategy.

Search strategy	
	• PubMed
Academic databases searched	• Science Direct
	• Google Scholar
	• Journals papers
Targeted items	• Workshop papers
	• Conference papers
	• Non-academic papers
	• Titles
Searched applied to	• Abstracts
	• Keywords (RT, hypo-fractionation RT, immune system, anti- tumour CD8+ T, Infiltration of CD8+ cytotoxic T-cells, tumour-specific, monocytic myeloid-derived suppressor cells (M-MDSCs), immune stimulation, RT-schedule, RT- dose, RT-fraction, and clinical outcome)
Language	• English
Publication periods	• Published between 2010 and 2024
	• RT-induced immune stimulation
Outcomes	• Improves bystander and Abscopal effects
	• Clinical outcomes, such as treatments and immune-related side effects.

### 2.2 Selection (inclusion and exclusion) criteria

The titles and abstracts of relevant studies were evaluated for their contents, ensuring adherence to this systematic review article’s inclusion and exclusion criteria. Inclusion criteria were (I) the studies investigating RT-induced immune stimulation; (II) the studies investigating immune cells such as CD8+ cytotoxic T-cells, regulatory T-cells (Tregs), and M-MDSCs after using HFRT; (III) the studies monitoring optimal RT-type, RT-dose, RT-fraction, RT-schedule, (IV) the studies investigating AE after using HFRT, time to AE, and site of AE; (IV) the studies recorded patient’s characteristics and association with the clinical outcome; and (V) the studies analysed the association of HFRT-induced immune stimulation and improved clinical outcomes including complete response (CR), partial response (PR) and stable disease (SD).

The exclusion criteria for this systematic review were (I) editorials, (II) case reports, (III) studies did not have primary data, (IV) studies did not report bystander effect (BE) and AE following HFRT in metastatic disease, (V) studies monitoring RT with Immunotherapy/Chemotherapy combination, (VI) studies which were not written in English, and (VII) did not have full text available. The articles that fulfilled the inclusion criteria were shortlisted, and the primary characteristics are summarised in **Table 2**.

**Table 2:** The main clinical characteristics of the included studies.

Authors, year	Pat. no.	Tumour type	Biopsy sample	HFRT schedule/no. fractions	Total dose (Gy)
Preclinical studies					
Filatenkov et al. (2015) (5)	14 mice	Colon tumours	CT26 and MC38 Cell lines	30 Gy × 1 fr	30 Gy
Markovsky et al. (2019) (32)	NA	Breast cancer and Lung cancer	67NR murine and LLC mouse model	3.5 Gy/minute	10 Gy
Kim et al. (2023) (33)	NA	Lung metastasis	FSaII, CT-26, and 4T1 cells	20 Gy × 1 fr and 10 Gy × 2 frs	20 Gy
Frey et al. (2017) (34)	NA	Colon Cancer	CT26 cells	5 Gy × 2 frs	10 Gy
Zhao et al. (2022) (36)	NA	Lung Cancer	Lewis lung carcinoma (LLC) cells	3.7 Gy × 4 frs, 4.6 Gy × 3 frs, 6.2 Gy × 2 frs, and 10 Gy × 1 fr	20Gy
Schaue et al. (2012) (38)	NA	Melanoma	B16-OVA murine	15 Gy × 1 fr and 7.5 Gy × 2 frs	15 Gy
Grapin et al. (2019) (39)	NA	Colon Cancer	CT26 cells	2 Gy × 18 frs 8 Gy × 3 frs and 16.4 Gy × 1 fr	36 Gy 24 Gy 16.4 Gy
Clinical studies					
Zhang et al. (2017) (35)	6	Non-small-cell lung cancer	Blood	48 Gy × 8 frs or 48 Gy × 6 frs	48 Gy
Muraro et al. (2017) (37)	21	Breast Cancer	Blood	10 Gy × 3 frs	30 Gy
McGee et al. (2018) (40)	31	Lung, Liver, Adrenal, Brain, Bone, and Other organs	PBMC and serum (pre- and 1–2 weeks post-SAR)	1–5 frs SBRT or 1–10 frs HCRT	NA
Tubin et al. (2019) (42)	23	Lung = 16 Kidney = 3 Skin = 2 Prostate = 1 Unknown = 1	NA	10–12 Gy × 1–3 frs	10–12 Gy
Tubin et al. (2019) (41)	60	Non-small cell lung cancer	NA	10 Gy × 3 frs	30 Gy

RT, radiotherapy; NA, not available; PD1, Programmed cell death protein 1; LLC, Lewis lung carcinoma; HFRT, hypo-fractionated RT; Gy, gray; no., number; frs, fractions; SBRT, stereotactic body radiation therapy; SRS, stereotactic radiosurgery; and HCRT, hypo-fractionated conformal radiotherapy.

### 2.3 Data extraction and quality assessment method

The data were extracted from selected studies by two authors. The extracted data included (I) general information (first author, publication’s year, method of patient recruitment, and sampling methods); (II) clinical characteristics (T-stage, age, treatment option, RT-type, RT-dose, RT-fraction, and RT-schedule); (III) T-cell response following HFRT and (IV) clinical outcomes (time to AE, site of AE, biochemical recurrence, side effects of RT or RT-induced toxicity, treatment response (CR and PR), tumour control, PFS and OS).

The study’s quality was assessed using the PROBAST (Prediction Model Risk of Bias Assessment Tool), which evaluates the applicability and risk of bias in diagnostic tests (30). To address discrepancies in interpretation, two assessors jointly assessed one article first. Articles were then scored for each study, and section deficiencies were noted for further discussion. The relevant published articles were retrieved and imported into an Endnote X21 database (31). Analogous articles were identified and deleted using the Endnote’s duplicate function. We considered studies only describing multivariable-adjusted hazard ratios (aHR). Moreover, we excluded studies that reported crude or unadjusted outcome measures between patients treated with HFRT.

## 3 Results

### 3.1 Systematic review analysis

**Figure 1:**
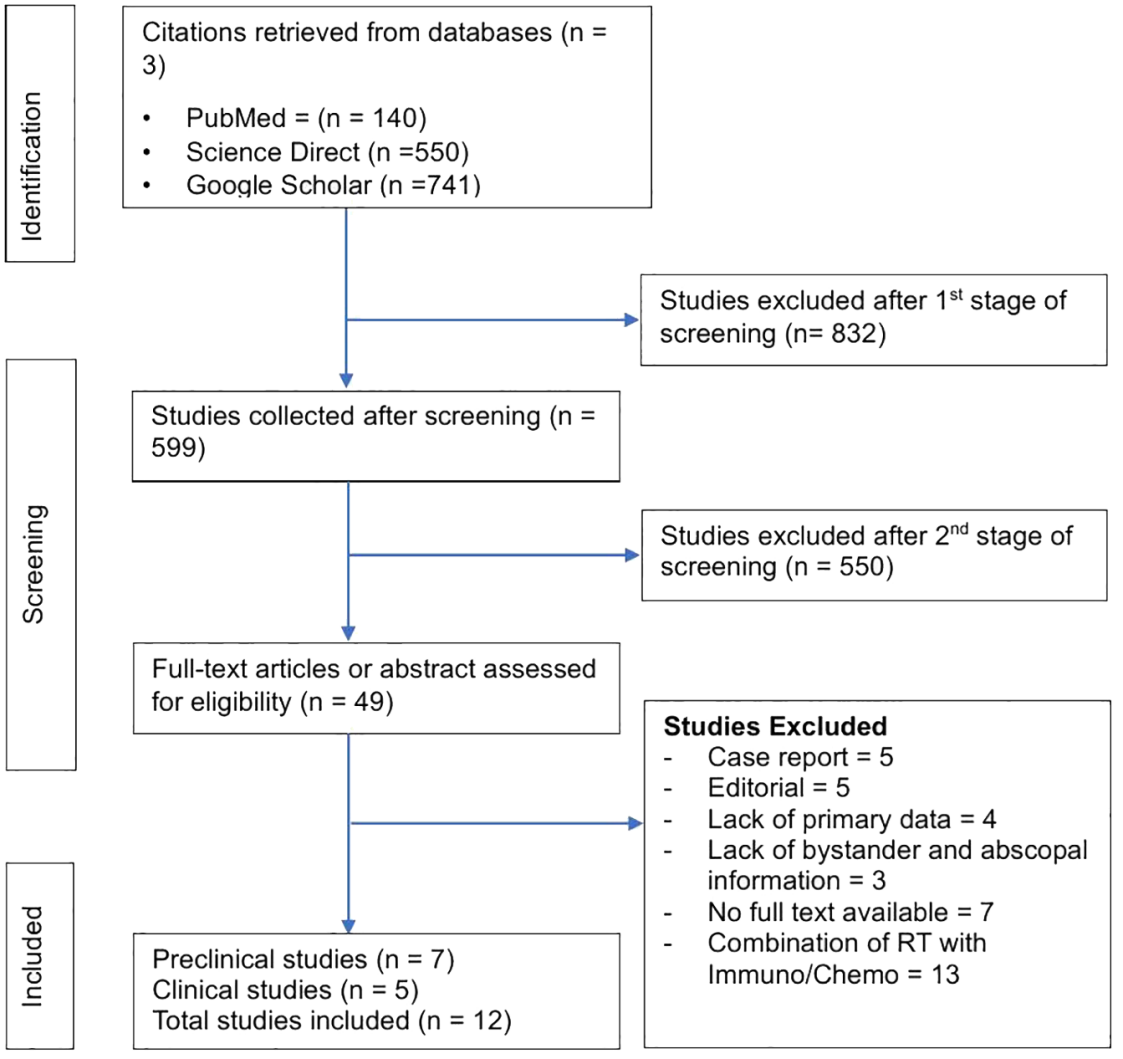
Representation of the PRISMA workflow for selecting studies.

The literature search identified 1431 preclinical and clinical studies: 140 from PubMed, 550 from Science Direct, and 741 from Google Scholar, respectively. Of these 1431 studies, 832 were excluded after reviewing the titles and abstracts, and 599 were selected at the first screening stage. At the second screening stage, 550 studies were removed after full-text examination, and 14 were selected. Furthermore, 49 studies were assessed for eligibility, and 34 studies were removed for the following reasons: (1) case report = 5; (2) editorial = 5; (3) lack of present primary data = 4; (4) lack of bystander and abscopal information = 3; (5) no full text available = 7; (6) studies investigating RT effect in combination with Immunotherapy/Chemotherapy = 13. After eligibility consideration, 12 (7 = preclinical and 5 = clinical) studies were selected. **Figure 1** shows our literature search and selection strategy as a flowchart.

### 3.2 T-cell response following HFRT

To evaluate the potential immunogenic modulation of HFRT at the optimal schedule, 10 of the 12 selected studies have observed T-cell infiltration to the tumour environment following HFRT (**Table 3**) (5, 32–40).

**Table 3:** Studies reported tumour-infiltrating CD8+ T-cell response in cancer patients following HFRT.

Authors, year	Country	Sample	Treatment option	RT dose/no. fractions	*P* - values	T-cell response observed
Preclinical studies						
Filatenkov et al. (2015) (5)	USA	CT26 and MC38 Cell lines	SABR	30 Gy × 1 fr	NA	Yes
Markovsky et al. (2019) (32)	USA	67NR murine, Lung Carcinoma (LLC) mouse model	SFR	10Gy × 3.5 Gy/minute	NA	Yes
Kim et al. (2023) (33)	Korea	FSaII, CT-26, and 4T1 cells	HFRT	20 Gy × 1 fr and 10 Gy × 2 frs	NA	Yes
Frey et al. (2017) (34)	Germany	CT26 cells	HFRT	5 Gy × 2 frs	NA	Yes
Zhao et al. (2022) (36)	China	Lewis lung carcinoma (LLC) cells	HFRT	3.7 Gy × 4 frs, 4.6 Gy × 3 frs, 6.2 Gy × 2 frs, and 10 Gy × 1 fr	*P* < 0.05	Yes
Schaue et al. (2012) (38)	USA	B16-OVA murine	SFR	15 Gy × 1 fr and 7.5 Gy × 2 frs	NA	Yes
Grapin et al. (2019) (39)	France	CT26 cells	HFRT	2 Gy × 18 frs 8 Gy × 3 frs and 16.4 Gy × 1 fr	*P* < 0.0011	Yes
Clinical studies						
Zhang et al. (2017) (35)	China	Blood	SBRT	48 Gy × 8 frs or 48 Gy × 6 frs	*P* = 0.0143	Yes
Muraro et al. (2017) (37)	Italy	Blood	SBRT	10 Gy × 3 frs	NA	Yes
McGee et al. (2018) (40)	USA	PBMC and serum (pre- and 1–2 weeks post-SAR)	SAR	1–5 frs SBRT or 1–10 frs HCRT	NA	Yes

NA, not available; RT, radiotherapy; Gy, gray; no., number; frs, fractions; LLC, Lewis lung carcinoma; HFRT, hypo-fractionated RT; SBRT, stereotactic body radiation therapy; SABR, stereotactic ablative body irradiation; SFR, Single fraction radiotherapy.

Out of 10, 7 preclinical studies reported an increased infiltration of T-cells to the tumour microenvironment after HFRT (5, 32–34, 36, 38, 39). For example, Filatenkov et al. reported that the unirradiated tumour with HFRT contained approximately 19% CD8+ T-cells, the irradiated tumour contained approximately 70% at day 35, and the percentage of MDSCs decreased after day 24 (5). In addition, there was a trend toward an increase in CD8+ T-cells in both irradiated and non-irradiated parts of the tumour seven days post-10 Gy RT (32). Kim et al. reported that five-day spacing was more effective than a one-day interval in enhancing anti-tumour immunity via activating the CD8+ T-cells and suppressing the M-MDSCs (33).

Some studies have demonstrated that optimal RT dose and fraction can cause immunologic effects and increased CD8+ T-cell infiltration in the tumour microenvironment (34, 36, 38, 39). Fray et al. stated that on day 8, more cytotoxic T-cells and a lower percentage of Tregs (CD4+/CD25+/FoxP3+) were identified in the irradiated tumours using irradiation two fractions × 5 Gy (34). In addition, an increase in CD8+ T-cells concentration was observed from 48 h to 3 weeks after HFRT in 4.6 Gy × 3 fractions and 6.2 Gy × 2 fractions (p < 0.05), but not in 3.7 Gy × 4 fractions and 10 Gy × 1 fraction (36). A single HFRT dose of 15 Gy increased CD8+ T-cell responses and decreased Tregs (38). The increased proportion of CD8+ T-cells was noticed on day seven after the first HFRT session in the 1 Fraction × 16.4 Gy group (p = 0.002), 3 fractions × 8 Gy group (p < 0.001), and in the 18 fractions × 2 Gy group (p < 0.001); versus 1.4% ± 0.3% in the control group (39).

In the clinical study group, three studies also stated the effect of HFRT on T-cell infiltration in cancer patients (35, 37, 40). Zhang et al. demonstrated that HFRT increased the frequency of CD8+ T-cell infiltration but decreased the frequency of inhibitory Tregs (35). Moreover, Muraro et al. also identified that half of the patients showed increased numbers of activated natural killer (NK) cells and T-cells (CD4+ and CD8+) immediately after the first dose of SBRT (37). Additionally, activated CD25+ CD4+ memory T-cells and CD25+ CD8+ memory T-cells increased following SAR to parenchymal sites, not bone or brain (40).

### 3.3 Tumour control following HFRT

To evaluate the efficacy of HFRT delivered in various schedules, 6 out of 12 selected preclinical and clinical studies tested the HFRT schemes with several-day intervals to control tumour growth (**Table 4**) (33, 34, 36, 38, 39, 41).

**Table 4:** Studies reported tumour control following HFRT.

Authors and Year	Country	Sample	Study endpoint	*P* - values	Tumour control observed
Preclinical studies					
Kim et al. (2023) (33)	Korea	FSaII, CT-26, and 4T1 cells	Tumour control	One-day interval RT (P = 0.641), Five-day interval RT (*P* = 0.029), and Seven-day interval RT (*P* = 0.043)	Yes
Frey et al. (2017) (34)	Germany	CT26 cells	Tumour control	NA	Yes
Zhao et al. (2022) (36)	China	Lewis lung carcinoma (LLC) cells	Tumour control	*P* < 0.01	Yes
Schaue et al. (2012) (38)	USA	B16-OVA murine	Tumour control	NA	Yes
Grapin et al. (2019) (39)	France	CT26 cells	Tumour control	NA	Yes,
Clinical studies					
Tubin et al. (2019) (41)	Austria	NA	Tumour control	NA	Yes

RT, radiotherapy; NA, not available; LLC, Lewis lung carcinoma; HFRT, hypo-fractionated RT; Gy, gray; no., number; frs, fractions; SBRT, stereotactic body radiation therapy; SRS, stereotactic radiosurgery; and HCRT, hypo-fractionated conformal radiotherapy.

In the preclinical studies group, 5 out of 6 reported tumour control after HFRT (33, 34, 36, 38, 39). Kim et al. reported that tumour growth delays by a five-day interval RT (p = 0.0293) or a seven-day interval RT (p = 0.0434) were more significant than those by a one-day interval (p = 0.6413) (33). Moreover, tumour growth was significantly delayed in the mice irradiated with 2 fractions × 5 Gy in a 4-day interval (34).

To evaluate the tumour control at different RT schedules, Zhao et al. reported that tumour growth was considerably delayed in the 6.2 Gy × 2 fractions group compared with the control group (p < 0.01) (36). Furthermore, the group receiving local single-dose HFRT at 7.5 and 15 Gy showed significant tumour control, whereas the group receiving 5 Gy had a minimal effect (38). In addition, Grapin et al. monitored the tumour’s growth with 18 fractions × 2 Gy and 3 fractions × 8 Gy regimens and found the most extended tumour growth delay compared to 1 fraction × 16.4 Gy (39).

On the other hand, only one clinical study reported that the bulky tumour control rate was 95% for the SBRT groups compared with 20% in the other two groups (41).

### 3.4 Consequent vaccine-like effect following HFRT

BE, or AE effect of HFRT, is a rare and unpredictable outcome encountered during the metastatic treatment, where tumour regression is observed to be distant from the irradiated volume. Eight previous preclinical and clinical studies have reported AE and clinical outcomes at optimal HFRT schedules (**Table 5**) (5, 32, 35–37, 40–42).

**Table 5:** Studies reported vaccine-like effects following HFRT.

Authors and Year	Country	Sample	Treatment option	Study endpoints	P - values	Response rate observed
Filatenkov et al. (2015) (5)	USA	CT26 and MC38 Cell lines	SABR	CR	NA	Yes
Markovsky et al. (2019) (32)	USA	67NR murine, Lung Carcinoma (LLC) mouse model	SFR	AE	NA	Yes
Zhao et al. (2022) (36)	China	Lewis lung carcinoma (LLC) cells	HFRT	OS	NA	Yes
Clinical studies						
Zhang et al. (2017) (35)	China	Blood	SBRT	OS	NA	Yes
Muraro et al. (2017) (37)	Italy	Blood	SBRT	PFS	NA	Yes
McGee et al. (2018) (40)	USA	PBMC and serum (pre- and 1–2 weeks post-SAR)	SAR	AE	NA	Yes
Tubin et al. (2019) (41)	Austria	NA	SBRT	OS CSS BE SE Symptom control	*P* = 0.099 *P* = 0.049 NA NA *P* = 0.018	Yes
Tubin et al. (2019) (42)	Austria	NA	SBRT	BE AE OS PFS	NA NA NA NA	Yes

RT, radiotherapy; Gy, gray; no., number; frs, fractions; LLCs, Lewis lung carcinoma; AE, abscopal effect; BE, bystander effect; PR, partial response; CR, complete response; OS, overall survival; PFS, progression-free survival; CSS, cancer-specific survival; ORR, objective rate response; HFRT, hypo-fractionated RT; SBRT, stereotactic body radiation therapy; SABR, stereotactic ablative body irradiation; SFR, Single fraction radiotherapy.

NA, Not available.

In the preclinical studies group, three studies have observed immunological effects and response rates following the use of HFRT. For example, 13 of the 14 mice achieved complete remissions when treated with 30 Gy, while 3 of 5 developed complete tumour remissions when the HFRT dose was specified at 20 Gy (5). Eight 67NR models (35%) experienced a significant AE after partial irradiation with a single dose of 10 Gy (32). Another preclinical study also stated that those treated with 6.2 Gy × 2 fractions showed a noteworthy improvement in OS compared to the control group (36).

In the clinical studies group, Kim et al. observed a better OS in patients treated with HFRT regimens of 48 Gy × 6 fractions or 48 Gy × 8 fractions, which activate the immune system three weeks after treatment (35). The patients showed increased numbers of activated natural killer (NK) cells immediately after the first SBRT dose, showing better PFS (37). Authors from another study have identified an AE in lung and liver cancer patients treated with 1–5 fractions of SBRT or 1–10 fractions of HCRT, but it was not observed in bone and brain (40).

Moreover, Tubin et al. observed AE in 45% (9/20) of patients treated with SBRT (41). They also observed that SBRT was more likely to improve survival OS rates (p = 0.099), cancer-specific survival (CSS) (p = 0.049) and PFS rates (p = 0.003) (41). Another Tubin study reported significant BE and AE response rates of 96% (22/23 patients) and 52% (12/23 patients), and improved OS 70% (16/23) and PFS 87% (20/23) rates, respectively, in patients whose bulky tumours were partially irradiated (42).

## 4 Discussion

Though RT has long been used in cancer therapy, it has a history of immunosuppressive side effects. Researchers believe that lymphopenia can result from localised RT, which includes radiation to the chest or central nervous system (43, 44). The leading causes of this are the radiation exposure of the bloodstream and the inherent radiation sensitivity of immune cells, even at low radiation doses (<1 Gy) (43–45). Although radiation has long been believed to suppress the immune system, there is a bunch of evidence showing that radiation can, under certain conditions, actually increase the immune system’s ability to fight cancer (5, 27, 32–41, 46–48).

Established tumour cells often lose their capacity to present antigens through various genetic and epigenetic mechanisms, enabling them to avoid the immune system. Radiation’s direct cytotoxic effects may cause the release of tumour-specific antigens, which can then prompt antigen-presenting cells to trigger a T-cell immune response (49). Although dendritic cells can present tumour antigens to T cells, the successful activation of tumour antigen-specific T-cell immunity requires additional danger signals to enhance T-cell activation (49). Therefore, during radiation-induced cell death, both tumour antigen release and presentation are improved, helping to activate an immune response (50). These specific events following radiation-induced tumour cell killing have led to the concept of utilising RT as a method of *in situ* vaccination” (51, 52).

Considering the increasing evidence that underlying anti-immune responses may be essential in eradicating certain tumours with SBRT, investigations have been conducted to delineate optimal radiation schedules for maximising anti-tumoural immunity in animal models (53, 54). Marciscano et al. extensively reviewed past studies on the optimal dose and fractionation schedule for increasing anti-tumoural immunity (55). Bae et al. reported that three days of fraction intervals significantly decreased gastrointestinal complications without impairing the tumour control rate of SABR in hepatocellular carcinoma (14). Moreover, using immunological hot and cold tumours, researchers also compared anti-tumoural immunity exposed to two fractions of irradiation administered on consecutive days or at intervals of 5 days in the mouse model (33).

Furthermore, when radiation is administered at moderate or higher dose fractions, local RT can activate CD8+ cytotoxic T-cells involved in both local and systemic tumour control (abscopal) (24, 46, 56). Therefore, in previous studies, RT with 3 to 5 doses of <10 to 12 Gy appears particularly immunogenic (11, 38, 57–59). Some earlier studies revealed that hRT elevates CD8+ concentration between days 5 and 8 after hRT (34, 60, 61). Filatenkov et al. reported that irradiation with 1 Fraction × 30 Gy was curative and induced protective CD8+ T-cell-mediated immunity (5). A similar protracted schedule (4 fractions × 5 Gy over 14 days) failed to locally control B16 melanoma tumours expressing a model antigen with a low total dose of RT and large inter-fraction intervals; however, a single 20 Gy fraction did so (46). Moreover, SRS with a single dose of at least 30 Gy has been suggested to be more effective than daily fractionated radiation (2, 6).

Several researchers have previously reported substantial immune effects and tumour reduction/cure through selective and time-dependent RT, which targets the immune system instead of the tumour (62–64). The effectiveness of these methods depended on the ability to determine when Tregs were dividing synchronously and periodically during cell division (65). At this brief window in time (mitosis), the Tregs were highly sensitive to selective ablation, thus mitigating or removing their homeostatic immunosuppressive effects on other tumour-specific immune cells not in mitosis at that specific time point (64). Due to the tumour’s underlying immunology, RT may evolve towards more “immunologically relevant” schedules to break tumour tolerance locally and systemically (66, 67).

Contrary to the results of RT studies, some studies, in combination with immunotherapy, found no evidence of AE and response rate after using HFRT (68, 69). For example, McBride’s and Kim et al. studies found no evidence of AE and improved clinical outcomes by adding SBRT to nivolumab and Nivolumab plus ipilimumab in patients with metastatic head and neck squamous cell carcinoma (HNSCC) and Advanced Merkel Cell Carcinoma, respectively (68, 69). The small sample size may have contributed to the lack of evidence of an additional benefit or support for AE with the addition of SBRT, as mentioned in these clinical trials. Some previous studies have shown potential therapeutic benefits with systemic therapies given at the right time to selectively ablate synchronously dividing suppressor T cells (now called Regulatory T Cells) while sparing the effector T cells (63, 70, 71). Therefore, it suggests that the timing of immunotherapy and RT may play a role in treatment efficacy via immune modulation. We believe that additional investigation is warranted to determine the optimal RT dose and timing, immunotherapeutic agent, and large patient cohort to fully evaluate the potential of the AE on the response rate.

## 5 Conclusions

Our systematic review data revealed that HFRT with an optimal regimen can induce the activation of T lymphocytes while simultaneously reducing the frequency of Tregs. These studies also suggested that optimal dosages and fractions of HFRT induce immune response. However, it should be further explored to provide clinicians with information that would be valuable when making decisions regarding patient care. This strategy may increase cancer patients’ response rate to treatment, lower the cost and length of treatment and lower thir chance of developing immune-related side effects and general toxicity after receiving chemotherapy and immunotherapy.
